# Health-Related Quality of Life and Treatment Satisfaction of Patients with Malignant IDH Wild-Type Gliomas and Their Caregivers

**DOI:** 10.3390/curroncol31100459

**Published:** 2024-10-14

**Authors:** Anna Fischl, Michael Gerken, Patricia Lindberg-Scharf, Tareq M. Haedenkamp, Katharina Rosengarth, Andrea Hillberg, Martin Vogelhuber, Ingrid Schön, Martin Proescholdt, Tommaso Araceli, Michael Koller, Anne Herrmann, Oliver Kölbl, Tobias Pukrop, Markus J. Riemenschneider, Nils Ole Schmidt, Monika Klinkhammer-Schalke, Ralf Linker, Peter Hau, Elisabeth Bumes

**Affiliations:** 1 Department of Neurology and Wilhelm Sander-NeuroOncology Unit, Regensburg University Hospital, 93053 Regensburg, Germany; anna.fischl@ukr.de (A.F.); tareq.haedenkamp@ukr.de (T.M.H.); ralf.linker@ukr.de (R.L.); peter.hau@ukr.de (P.H.); 2 Center for Quality Assurance and Health Services Research, University of Regensburg, 93053 Regensburg, Germany; michael.gerken@ukr.de (M.G.); patricia.lindberg-scharf@klinik.uni-regensburg.de (P.L.-S.); monika.klinkhammer-schalke@ukr.de (M.K.-S.); 3 Department of Neurosurgery, Regensburg University Hospital, 93053 Regensburg, Germany; katharina.rosengarth@ukr.de (K.R.); martin.proescholdt@ukr.de (M.P.); tommaso.araceli@ukr.de (T.A.); nils-ole.schmidt@ukr.de (N.O.S.); 4 Department of Internal Medicine III, Regensburg University Hospital, 93053 Regensburg, Germany; andrea.hillberg@ukr.de (A.H.); martin.vogelhuber@ukr.de (M.V.); ingrid.schoen@ukr.de (I.S.); tobias.pukrop@ukr.de (T.P.); 5 Center for Clinical Trials, Regensburg University Hospital, 93053 Regensburg, Germany; michael.koller@ukr.de; 6 Department of Epidemiology and Preventive Medicine/Medical Sociology, University of Regensburg, 93053 Regensburg, Germany; anne.herrmann@ukr.de; 7 Department of Radiotherapy, Regensburg University Hospital, 93053 Regensburg, Germany; o.koelbl@ukr.de; 8 Department of Neuropathology, Regensburg University Hospital, 93053 Regensburg, Germany; markus.riemenschneider@ukr.de

**Keywords:** glioma, IDH wild-type, psycho-oncology, health-related quality of life, HR-QoL, satisfaction

## Abstract

(1) Background: Clinical aspects like sex, age, Karnofsky Performance Scale (KPS) and psychosocial distress can affect the health-related quality of life (HR-QoL) and treatment satisfaction of patients with malignant isocitrate dehydrogenase wild-type (IDHwt) gliomas and caregivers. (2) Methods: We prospectively investigated the HR-QoL and patient/caregiver treatment satisfaction in a cross-sectional study with univariable and multiple regression analyses. Questionnaires were applied to investigate the HR-QoL (EORTC QLQ-C30, QLQ-BN20) and treatment satisfaction (EORTC PATSAT-C33). (3) Results: A cohort of 61 patients was investigated. A higher KPS was significantly associated with a better HR-QoL regarding the functional scales of the EORTC QLQ-C30 (*p* < 0.004) and a lower symptom burden regarding the EORTC QLQ-BN20 (*p* < 0.001). The patient treatment satisfaction was significantly poorer in the patients older than 60 years in the domain of family involvement (*p* = 0.010). None of the investigated aspects showed a significant impact on the treatment satisfaction of caregivers. (4) Conclusions: We demonstrated that in patients with IDHwt gliomas, the KPS was the most important predictor for a better HR-QoL in functional domains. Data on the HR-QoL and treatment satisfaction in patients with IDHwt gliomas and their caregivers are rare; therefore, further efforts should be made to improve supportive care in this highly distressed cohort.

## 1. Introduction

Malignant isocitrate dehydrogenase wild-type (IDHwt) gliomas are incurable primary tumors of the brain [1] and cause a variety of symptoms in affected patients [2]. In addition to neurological symptoms, changes in cognition and personality are common and should be diagnosed and treated early [3]. To support patients in the best possible way to meet their needs, it is important to evaluate the health-related quality of life (HR-QoL) and treatment satisfaction regularly.

Investigating the HR-QoL is a mainstay in the integration of patient-reported outcomes [4,5] and covers the social functioning, psychological well-being, daily activity level and physical functioning [6]. The EORTC QLQ-C30 questionnaire was validated in cancer patients to assess the HR-QoL and is commonly used in glioma patients [7]. Additionally, to compile brain-tumor-specific symptoms, the EORTC QLQ-BN20 module questionnaire was developed and validated [8]. Both questionnaires are regularly used to investigate the HR-QoL in patients with brain tumors [9].

General clinical aspects like sex, age at diagnosis and performance status interact with HR-QoL. External tools to evaluate the performance status of a patient are the Karnofsky Performance Scale (KPS) and the Eastern Cooperative Oncology Group (ECOG) scale [10,11].

A poorer HR-QoL of female patients, compared with male patients, was observed in many tumor entities [12,13,14,15,16,17]. It was also demonstrated that female glioma patients have worse physical functioning and role functioning [18].

In other tumor entities, older patients were observed to report a poorer global HR-QoL, physical functioning [15,19,20] and role functioning [12]. An analysis of the correlation between the age at diagnosis and HR-QoL revealed that patients with a high-grade glioma aged ≥ 65 years had a poorer global health status and poorer physical and cognitive functioning compared with younger patients [18,21]. A higher symptom burden was revealed regarding visual disorders [21] and motor dysfunction [18,21] in glioma patients ≥ 65 years.

In addition, the performance status interacts with the HR-QoL: glioma patients with a worse ECOG status or KPS ≤ 70% have a poorer global health status, physical functioning and role functioning in the period up to first progression [18,21]. An inconsistent effect was demonstrated for the symptom burden: patients with a high-grade glioma and a KPS ≥ 70% had a higher symptom burden regarding future uncertainty, visual disorder, motor dysfunction and communication deficits [21]. In contrast, Coomans et al. demonstrated that a poorer ECOG status was a negative predictor for a greater symptom burden regarding motor dysfunction during the progression-free period [18].

Despite the proven impact of these clinical factors on HR-QoL, there are so far no trials that addressed this topic, particularly in patients with a malignant IDHwt glioma.

Not only physical conditions but also psychosocial distress [22] can impact the HR-QoL. Brain tumor patients commonly suffer from distress [23,24], and therefore, have a significantly reduced global HR-QoL [25]. Furthermore, higher levels of future uncertainty were found in brain tumor patients who were distressed [25].

A brain tumor diagnosis also negatively affects caregivers’ HR-QoL and treatment satisfaction [26]. Caregivers are usually less satisfied than patients [27] and poorer treatment satisfaction is associated with a poorer HR-QoL of patients [28]. Recent publications also demonstrate that caregiver and patient treatment satisfaction influence each other [29,30,31]. Additionally, a semi-structured interview revealed that caregivers have different demands than patients [32]. Therefore, evaluating their needs is also important.

Although patients with malignant IDHwt gliomas and their caregivers are often highly distressed and negatively affected in their HR-QoL, data on HR-QoL and treatment satisfaction are sparse. Furthermore, a review demonstrated that only 41% of the investigated studies chose quality of life as a secondary outcome and none of them as a primary outcome parameter [33]. We conducted this cross-sectional study at a large academic primary brain cancer center to gain more information on factors that correlate with the HR-QoL and treatment satisfaction in patients with IDHwt gliomas and their caregivers. In this analysis, we evaluated the differences in the HR-QoL and treatment satisfaction with respect to the age at diagnosis, sex, KPS and psychosocial distress.

## 2. Materials and Methods

### 2.1. Study Design

This study was designed as a cross-sectional study. Data on the HR-QoL and patient/caregiver treatment satisfaction were gathered from 1 November 2019 until 30 September 2020, with an interruption due to the COVID-19 pandemic from 23 March 2020 until 5 May 2020. After providing informed consent, paper-based questionnaires were given to patients and caregivers to prospectively evaluate the HR-QoL and treatment satisfaction at regular outpatient visits during the study period. If the patients and caregivers completed questionnaires at more than one time point, the most recent assessment date was chosen. The HR-QoL and treatment satisfaction were assessed regarding the independent factors of sex; age at diagnosis; KPS at last assessment of the HR-QoL/treatment satisfaction; and psychosocial distress at the first outpatient visit, as measured with the Hornheider Screening Instrument (HSI).

### 2.2. Study Population

Patients with a diagnosis of malignant IDHwt glioma who were registered between January 2014 and September 2020 in a local tumor registry and filed in our hospital data management system were included in this cross-sectional study. The main inclusion criteria were an age at diagnosis of 18 years or older, histology of malignant IDHwt glioma (IDHwt glioblastoma and IDHwt anaplastic astrocytoma according to the WHO classification of 2016 [34]), at least two on-site visits at the Brain Cancer Center at University Hospital Regensburg, being alive at the beginning of the study period (1 November 2019) and informed consent.

To investigate the caregivers’ satisfaction, the person who mainly took care of the patient at home was defined as the caregiver.

### 2.3. Sociodemographic, Clinical and Treatment Factors

The following sociodemographic and treatment factors were derived from the electronic medical records: sex; age at diagnosis; date of glioma diagnosis; histology and WHO grade according to the WHO classification of 2016 [34], which was valid during the study period; diagnosis of epilepsy at the first outpatient visit; psychosocial distress at the first outpatient visit (measured with the HSI); received psycho-oncological treatment; KPS at the last assessment of the HR-QoL; patient/caregiver treatment satisfaction; and time range between diagnosis and last assessment of HR-QoL/treatment satisfaction. The neurological functional status was evaluated using the Neurologic Assessment in Neuro-Oncology (NANO) [35] scale. Due to structural reasons, the assessment was incomplete and could therefore not be included in the analysis.

Information regarding the psycho-oncological treatment was collected during regular follow-up visits and at the request of psycho-oncologists.

### 2.4. Psycho-Oncological Need and Treatment

The psychosocial distress, and therefore, the need for psycho-oncological treatment was evaluated with the HSI [22]. The patients with a summary score ≥ 4 received at least one consultation with a dedicated clinical psycho-oncologist. The individual patient’s burden was addressed, and coping strategies were discussed during a psycho-oncological consultation. In most cases, cognitive behavioral therapy was applied [36].

### 2.5. Health-Related Quality of Life and Patient and Caregiver Treatment Satisfaction

The HR-QoL was assessed with the questionnaires EORTC QLQ-C30 [7] and a module for brain tumors EORTC QLQ-BN20 [8]; the patient and caregiver treatment satisfaction was assessed with EORTC PATSAT-C33 [37].

The EORTC QLQ-C30 questionnaire (version 3.0) was validated and translated into German [38]; this version was applied in our study. It contains 30 items, which are assessed as multi-item scales and single items. All together, they reflect the multidimensionality of the quality of life. The EORTC QLQ-C30 includes physical, role, cognitive, emotional and social functioning as functional scales. These are assessed as multi-item scales. Additionally, it contains three multi-item symptom scales (fatigue, pain, and nausea and vomiting) and a multi-item scale of global health and quality of life. Common physical symptoms of cancer disease, like dyspnea, appetite loss, sleep disturbance, constipation and diarrhea, are assessed as single items. Furthermore, possible financial difficulties caused by the disease and treatment are evaluated. The EORTC QLQ-C30 is validated in cancer patients and regularly used in glioma trials [7,39,40,41,42].

The EORTC QLQ-BN20 brain cancer module is an organ-specific addition to the EORTC QLQ-C30 to specifically address the disease- or treatment-related symptoms of brain cancer patients [8]. It consists of 20 questions, which include four multi-item scales to assess the symptoms of future uncertainty, visual disorder, motor dysfunction and communication deficit. Additionally, seven other common brain-tumor-specific symptoms are addressed as single items: headache, seizure, drowsiness, hair loss, itchy skin, weakness of legs and bladder control [8].

The EORTC PATSAT-C33 was validated in cancer patients [37]. It consists of 33 questions with seven multi- and five single-item scales. The multi-item scales evaluate the doctors’ technical skills, information exchange and affective behavior of the doctors, as well as information exchange, responsiveness, and affective behavior of the nursing staff and radiotherapy technicians. Additionally, the coordination and interaction with healthcare providers regarding the services and care organization are addressed. The single-item scales evaluate the satisfaction with family involvement, access to car parks and walkways, environment and overall care [37].

Questionnaires were defined as valid if more than half of the questions were answered, following the official regulations of the EORTC [43].

The EORTC QLQ-C30, QLQ-BN20 and EORTC PATSAT-C33 were analyzed using metric scores. We analyzed the functional scales of EORTC QLQ-C30, as they represent the multidimensional HR-QoL: physical functioning, role functioning, emotional functioning, cognitive functioning and social functioning. Additionally, the scale of the global health status was investigated. Questions concerning the functional status are answered on a four-point Likert scale with a range from “not at all” to “very much”, while the global health status is rated on a seven-point Likert scale with a range from “very poor” to “excellent”. For the standardization of these raw item scores, a linear transformation is used to change the raw item scores to scores from 0 to 100. A high score of the functional scales represents a high/healthy level of functioning and a high score of the global health status represents a high HR-QoL.

For EORTC QLQ-BN20, only the clustered symptom burden scales were analyzed: future uncertainty, visual disorder, motor dysfunction and communication deficit. Questions are answered on a four-point Likert scale with a range from “very poor” to “excellent”. The transformation in scores ranged from 0 to 100, the same as the EORTC QLQ-C30. A high score represents a high level of symptomatic burden.

For EORTC PATSAT-C33, scales relevant in clinical experience were statistically assessed: doctor/technical skills, doctor/information exchange, doctor/affective behavior, family involvement and overall care scale. These questions are answered with a five-point Likert scale with a range from “very poor” to “excellent”. The raw item scores were transformed linearly to a score that ranged from 0 to 100. A high score represents a high level of satisfaction with the care/perceived care quality.

### 2.6. Statistics

Pseudonymized data were recorded and analyzed in IBM SPSS (IBM SPSS Statistics for Windows, Version 25.0. Armonk, NY, USA: IBM Corp.). Continuous data were expressed as means, medians, standard deviations, minima and maxima. In the case of a normal distribution of the continuous variables, Student’s *t*-test was performed to compare the mean values. The Mann–Whitney *U* test was used for non-normal distributions. Categorical variables were described as absolute and relative frequencies. The independence between categorical variables was tested with Pearson’s chi-square test. If the sample size was too small, Fisher’s exact test was applied. The level of significance was set at *p* < 0.05.

The interference of clinical aspects, HR-QoL and treatment satisfaction was investigated with univariable and multiple linear regression analyses. We identified the following factors as possible confounders: sex, age at diagnosis, histology, diagnosis of epilepsy, psychosocial distress (measured with the HSI), psycho-oncological treatment, KPS, and time range between the diagnosis and the last assessment of the questionnaires. The sociodemographic and clinical aspects were chosen based on a literature review and clinical experience. The time range between the diagnosis and the last assessment was included due to the prospective inclusion of patients, and therefore, a variation in the time ranges between the diagnosis and the last assessment of the HR-QoL and treatment satisfaction data. Consequently, these possible confounders were applied in all the HR-QoL and treatment satisfaction models. The cut-off *p*-value in the univariable regression analysis was defined as *p* < 0.2, and all single variables that met this criterion were consequently included in the multiple linear regression analysis for adjustment. Therefore, the implemented confounders differed between the multiple regression analyses of each scale of the questionnaire. In the multiple linear regression analysis, the level of significance was set at *p* < 0.05.

### 2.7. Ethical and Regulatory Framework

This study was approved by the Regensburg University Institutional Ethics Review Board (vote no. 19-1375-101). Written informed consent and a data protection declaration were obtained from both the patients and caregivers, following German ethics and regulatory standards. The data protection concept of the Department of Neurology—Neuro-Oncology at Brain Tumor Center Regensburg, which acts in the framework of the European General Data Protection Regulation and relevant national legislation, was followed while processing the patient data.

## 3. Results

### 3.1. Patient Characteristics

In summary, 491 patients were selected by a data query at the regional brain tumor registry and screened for the inclusion criteria. At the initiation of this study, 61 (12.4%) of the preselected patients were alive, had a tumor histology of IDHwt glioblastoma or IDHwt anaplastic astrocytoma, had at least two on-site visits and consented to participate in this cross-sectional study (Figure S1).

All data drawn from the regional brain tumor registry and the electronic patient charts were complete. The demographic and clinical characteristics were distributed as shown in Table 1.

In our cohort of 61 patients, 33 (54.1%) patients were younger than 60 years at the first diagnosis and 31 (50.8%) patients were female (Table 1). A total of 24 (39.3%) patients had psychosocial distress (measured with the HSI) at the first outpatient visit and 29 (47.5%) received psycho-oncological treatment during the course of the disease (Table 1).

The time ranges between the date of the tumor diagnosis and the date of the last assessment of the HR-QoL and treatment satisfaction were assessed to correct for the treatment and prognostic effects (Table 1). Likewise, the KPS at the timepoint when the questionnaires were last gathered was investigated (Table 1). For both aspects, the results are exemplarily shown for the EORTC QLQ-C30 (Table 1).

The assessed questionnaires were filled in at different therapy stages: five (8.2%) patients completed the questionnaires after the initial surgery before starting a radiochemotherapy, one (1.6%) patient during radiochemotherapy and 11 (18.0%) patients during adjuvant chemotherapy after finalized radiotherapy. During the follow-up period, 19 (31.2%) patients were surveyed. Patients most often (n = 25, 41%) answered the questionnaires in the recurrence situation.

### 3.2. Distribution of HR-QoL and Patient/Caregiver Treatment Satisfaction Questionnaires

A total of 540 questionnaires were distributed, including 141 EORTC QLQ-C30, 140 EORTC QLQ-BN20, 136 EORTC PATSAT-C33 questionnaires for patients and 123 EORTC PATSAT-C33 questionnaires for caregivers. The valid completion rate was 141 (100%) for EORTC QLQ-C30 and 139 (99%) for EORTC QLQ-BN20, while 129 (95%) patients and 115 (93%) caregivers validly completed the EORTC PATSAT-C33 questionnaires (Figure S2).

### 3.3. Health-Related Quality of Life

We measured the HR-QoL with the core module EORTC QLQ-C30 and the brain-tumor-specific module QLQ-BN20 questionnaire.

**Figure 1:**
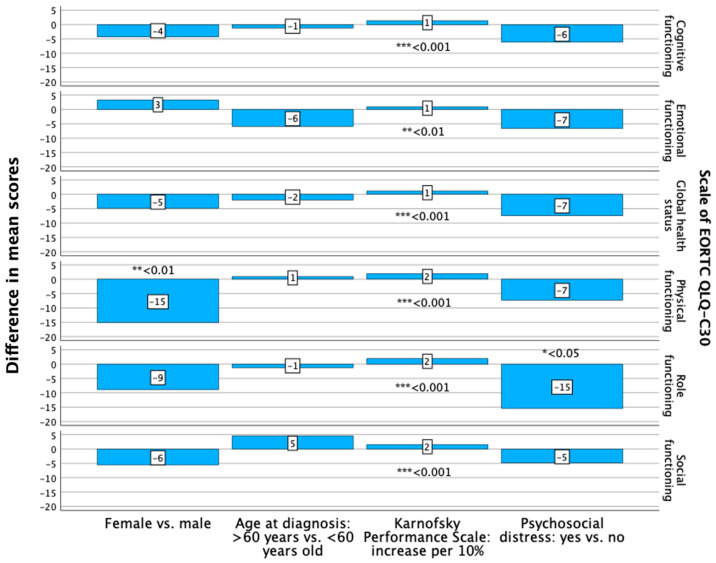
Differences in mean scores of the investigated scales of the EORTC QLQ-C30, analyzed with multiple regression analyses, regarding sex, age at diagnosis, Karnofsky Performance Scale and psychosocial distress. Significant differences are noted: * *p* < 0.05, ** *p* < 0.01, *** *p* < 0.001.

Female patients had a lower level of functioning regarding physical and role functioning (Figure 1). The difference between both groups was significant for the domain of physical functioning (*p* = 0.010; B: −15.166; 95% CI: lower: −26.508, upper: −3.824) (Table S1).

**Figure 2:**
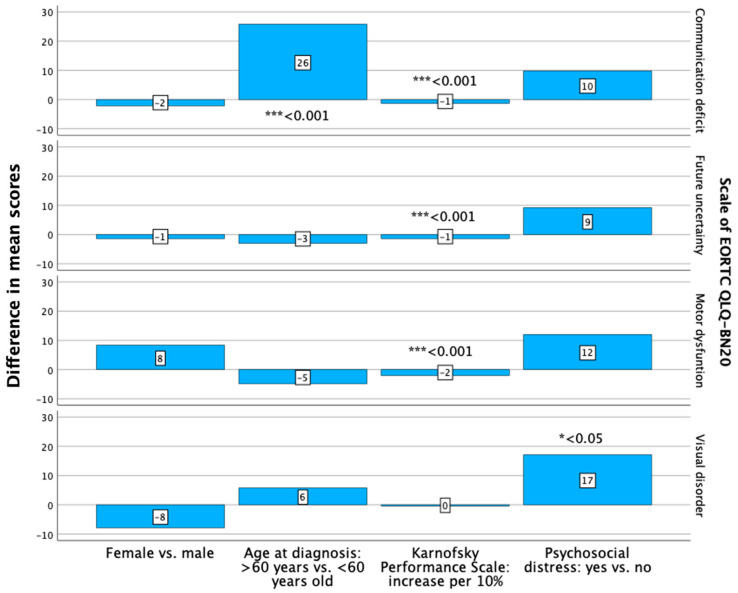
Differences in the mean scores of the investigated scales of the EORTC QLQ-BN20, analyzed with multiple regression analyses, regarding sex, age at diagnosis, Karnofsky Performance Scale and psychosocial distress. Significant differences are noted: * *p* < 0.05, *** *p* < 0.001.

Regarding the EORTC QLQ-BN20, the female patients had a higher symptom burden concerning the domain motor dysfunction (Figure 2). There were no significant differences in the investigated domains between the female and male patients (Figure 2).

The patients aged 60 years or older at diagnosis had a non-significantly higher level of functioning in two out of the five investigated domains (Figure 1) and a significantly (*p* < 0.001) higher symptom burden regarding communication deficits (B: 25.774; 95% CI: 11.198–40.350) (Figure 2, Table S2).

The patients with a superior KPS (meaning an increase in steps of 10%) had a significantly better HR-QoL and higher level of functioning regarding the global health status, physical, role, emotional, cognitive and social functioning, as assessed with the EORTC QLQ-C30 (Figure 1). The difference between the inferior and superior KPS was significant for all the investigated HR-QoL domains (*p* < 0.001/*p* = 0.004) (Figure 1, Table S1). The analysis of the EORTC QLQ-BN20 revealed that the patients with a superior KPS had a lower symptom burden regarding future uncertainty, visual disorder, motor dysfunction and communication deficit (Figure 2). The differences were significant (*p* < 0.001) in the domains of future uncertainty, motor dysfunction and communication deficit (Figure 2, Table S2).

Patients with psychosocial distress at the first outpatient visit had a poorer HR-QoL, as measured with the EORTC QLQ-C30, concerning the global health status and a lower level of functioning concerning role functioning, with a significant difference regarding the latter domain (*p* = 0.013; B: −15.475; 95% CI: lower: −27.487, upper: −3.462) (Table S1, Figure 1). The symptom burden regarding the EORTC QLQ-BN20 was higher in the patients with psychosocial distress concerning future uncertainty, visual disorders and motor dysfunction (Figure 2). The difference between patients with and without psychosocial distress was significant for the domain visual disorders (*p* = 0.019; B: 17.117; 95% CI: 2.970–31.264) (Table S2, Figure 2).

### 3.4. Patient and Caregiver Treatment Satisfaction

We assessed the patient and caregiver treatment satisfaction with the EORTC PATSAT-C33 questionnaire.

**Figure 3:**
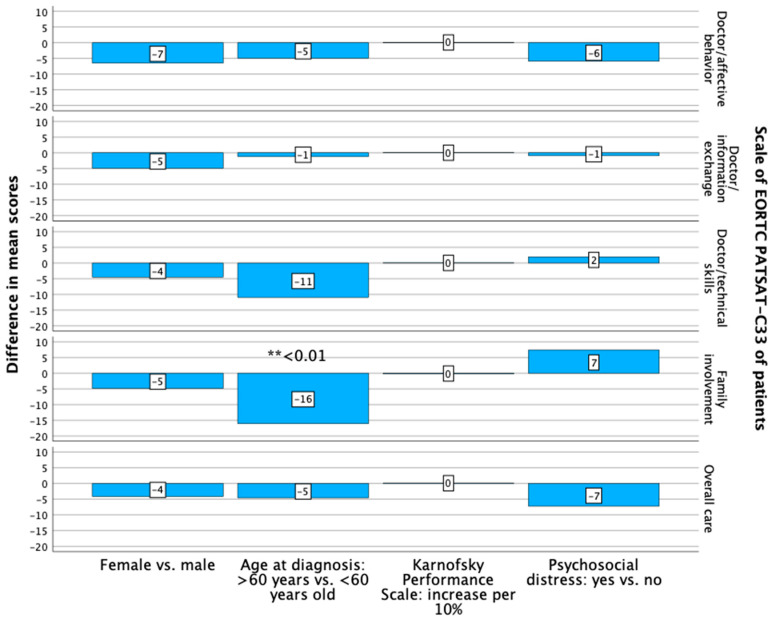
Differences in the mean scores of the investigated scales of the EORTC PATSAT-C33 of the patients, analyzed with multiple regression analyses, regarding sex, age at diagnosis, Karnofsky Performance Scale and psychosocial distress. Significant differences are noted: ** *p* < 0.01.

The female patients had a lower level of treatment satisfaction compared with the male patients, but the difference was not significant (Figure 3).

**Figure 4:**
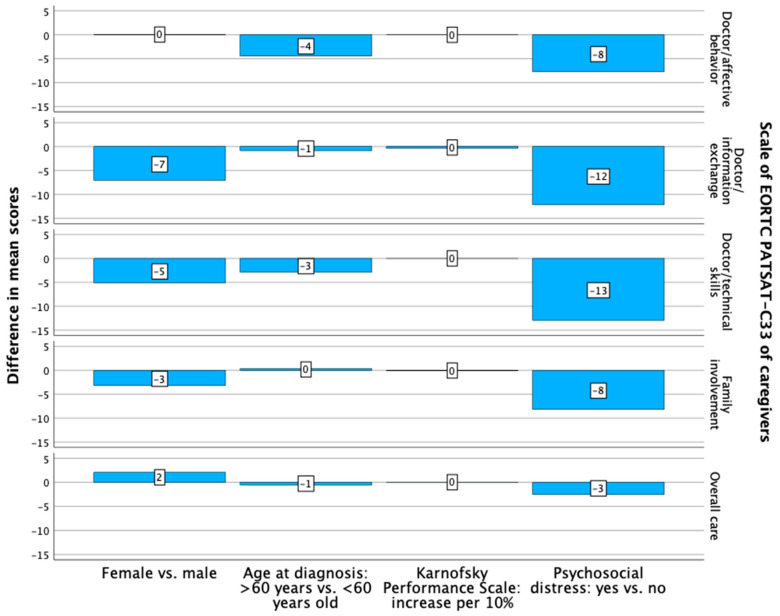
Differences in the mean scores of the investigated scales of the EORTC PATSAT-C33 of the caregivers, analyzed with multiple regression analyses, regarding sex, age at diagnosis, Karnofsky Performance Scale and psychosocial distress.

The caregivers of female patients had a higher, non-significant level of treatment satisfaction in two out of the five investigated domains (Figure 4).

The patients aged 60 years or older at diagnosis had a non-significantly lower level of treatment satisfaction concerning the technical skills of the doctors (Figure 3). A significantly lower level of satisfaction of these patients with treatment was found regarding family involvement (*p* = 0.010; B: −16.070; 95% CI: lower: −28.181, upper: −3.959) (Table S3, Figure 3). The caregivers of patients who were aged 60 years or older at diagnosis had a non-significantly lower level of treatment satisfaction in four out of the five investigated domains (Figure 4).

The patients with a superior KPS had a non-significantly, higher level of treatment satisfaction in four out of the five investigated domains (Figure 3). The caregivers of the patients with a superior KPS had a non-significantly lower level of treatment satisfaction with the information exchange with doctors (Figure 4).

The patients with psychosocial distress at their first outpatient visit had a non-significantly lower level of treatment satisfaction in three out of the five investigated domains (Figure 3). The caregivers of patients with psychosocial distress at the first outpatient visit had a lower level of treatment satisfaction regarding the technical skills of and information exchange with the doctors, without significant differences between the groups (Figure 4, Table S4).

## 4. Discussion

Data on the relationship between the HR-QoL and the treatment satisfaction in patients with malignant IDHwt gliomas are sparse and data that include caregivers are almost completely missing. Additionally, the HR-QoL as a primary or secondary outcome parameter is also scarce. The aim of this study was to assess the factors that correlate with the HR-QoL and treatment satisfaction of these patients and their caregivers. These data contribute to improving supportive care for this highly distressed cohort of patients with malignant IDHwt gliomas and their caregivers.

Supportive care provides an essential means of care, especially in patients with incurable cancers and their caregivers. Therefore, we prospectively evaluated the HR-QoL and treatment satisfaction in this cross-sectional clinical study in a homogenous cohort of patients with malignant IDHwt gliomas and their caregivers. Based on sociodemographic and clinical factors, differences in the HR-QoL and treatment satisfaction were investigated using univariable and multiple linear regression analysis.

Interestingly, we found statistically significant differences between the specific patient groups, namely, female vs. male, older vs. younger and superior vs. inferior functional states. The female patients had a poorer HR-QoL in some investigated domains and patients older than 60 years had a higher symptom burden regarding some assessed domains. Regarding a more favorable KPS, with increases in 10% steps, the patients had a better HR-QoL, higher level of functioning and a lower symptom burden in all the analyzed domains. The distressed patients had a lower level of role functioning, and in some assessed domains, a higher symptom burden. The analysis of treatment satisfaction of patients and caregivers revealed that the older patients had a lower level of treatment satisfaction regarding some investigated domains. Due to the high completion rate of all the questionnaires, the burden of filling in the questionnaires seemed to be acceptable.

### 4.1. Role of Sex in HR-QoL

First, the female patients had a lower level of physical functioning compared with the male patients. This finding fits very well with the published literature in brain tumor patients [18] and was also shown in female sarcoma patients [15]. Consistent with these findings, we demonstrated a non-significantly higher symptom burden regarding the domain motor dysfunction in female patients. In addition, one previous study revealed female sex as a significant predictor for an increase in symptom burden in the course of disease in glioma patients [18]. This could not be confirmed in our study due to the limited cohort size and the missing longitudinal follow-up in our study.

### 4.2. Role of Age in HR-QoL

Furthermore, we found that the patients 60 years or older had a non-significantly lower level of functioning regarding role, emotional and cognitive functioning. Published data confirm these findings: older patients have a somewhat poorer level of functioning compared with younger patients with high-grade gliomas [18,21], a finding that was verified in other tumor entities [12,15,19,20]. In our cohort, the symptom burden was significantly higher for patients aged 60 years or older in the communication deficit domain. This finding is not surprising as the HR-QoL is poorer in older patients and it fits well with the literature, where a higher symptom burden for older patients is described [18,21].

### 4.3. Role of Functional Status in HR-QoL

Next, the patients with a superior KPS, with increases in 10% steps, demonstrated a significantly higher level of functioning compared with patients with an inferior KPS. This finding was also observed in Renovanz et al., who demonstrated a higher level of functioning in high-grade glioma patients with a KPS ≥ 70% [21]. Furthermore, a poorer ECOG status was shown to be a predictor for the deterioration of the global health status and level of functioning during the course of a disease [12,18]. Additionally, it was demonstrated that the symptom burden regarding motor dysfunction increased over time when the ECOG status was poorer [18]. We detected the same correlation in our cohort: patients with a malignant IDHwt glioma and a superior KPS had a significantly reduced symptom burden regarding future uncertainty, motor dysfunction and communication deficit. However, other published data reveal opposing results: high-grade glioma patients had a higher symptom burden regarding future uncertainty, visual disorder, motor dysfunction and communication deficit if the KPS was at least 70% or higher [21]. To resolve these contradictory results, it may be speculated that at least some patients with a superior KPS perceive life-limiting disease and physical deficits as more restrictive.

### 4.4. Role of Psychosocial Distress in HR-QoL

In our cohort, the patients with a malignant IDHwt glioma with psychosocial distress had a significantly reduced level of role functioning. Although a malignant IDHwt glioma diagnosis is a life-limiting disease and patients frequently have high psychosocial distress, there have been no dedicated trials that have addressed this issue. There were few studies in other tumor entities that evaluated psychosocial distress with the HSI [44,45], where they observed that patients had a poorer global health status if they had psychosocial distress [44,45]. In one study with brain tumor patients, the distress thermometer and the hospital anxiety and depression scale were used and demonstrated a poorer HR-QoL in distressed patients [25]. Consistent with this study, distressed patients in our cohort also had a higher symptom burden.

### 4.5. Treatment Satisfaction in the Patient Cohort

The assessment of the treatment satisfaction of patients with malignant IDHwt gliomas revealed a significantly lower level of treatment satisfaction regarding the domain family involvement for patients aged 60 years or older. We could not find a significant correlation between other clinical aspects and the patients’ treatment satisfaction. A relationship between the patients’ HR-QoL and treatment satisfaction can be postulated. Hannon et al. demonstrated a correlation between a poor HR-QoL and reduced treatment satisfaction [27]. In addition, colorectal cancer patients who were less satisfied with their treatment had a reduced HR-QoL [28].

### 4.6. Treatment Satisfaction of Caregivers

Furthermore, we investigated the treatment satisfaction of the caregivers of the patients with a malignant IDHwt glioma who were strongly related to the patients and provided an important segment of patient care. Caregivers are not only key figures in the patient’s well-being, but they also influence treatment decisions and are often the main communication partner with healthcare professionals, especially if the patient has communication barriers, such as aphasia [46,47]. Severe psychosocial distress and reduced treatment satisfaction on the caregiver’s part have a negative impact on both the caregiver and the patient [30,31]. However, the treatment satisfaction of caregivers is often given little importance and is not sufficiently considered. Data on interventions in the context of the psychosocial distress of caregivers are still limited [48].

In our study, aspects like sex, KPS, age at diagnosis or psychosocial distress of patients revealed no significant correlation with the caregiver treatment satisfaction. We found, as a non-significant result, that the caregivers of patients with psychosocial distress at the first outpatient visit had a lower level of treatment satisfaction regarding the technical skills of and information exchange with the doctors compared with the caregivers of patients without psychosocial distress. A mutual influence of patients and caregivers was shown in other studies [30,31]. The severe symptom burden, reflected in distress, which, from the caregivers’ point of view, may be not adequately addressed by the doctors, may have contributed to a deterioration in the relationship between the caregiver and the doctor. This may have resulted in a decreased level of treatment satisfaction in the domains of information exchange with doctors and the technical skills of doctors. Further research is needed to clarify why these domains in particular are affected and how the needs of caregivers can be better addressed.

### 4.7. Limitations and Strengths

Our study had several limitations. First, this was a single-center study in a rural area. Results may differ for sites with a different sociodemographic background. However, the Regensburg Brain Cancer Center reflects the average quality parameters of the German certification system. Furthermore, the interpretation of treatment satisfaction results is not as easy due to the fact that treatment satisfaction can be influenced by many aspects. Therefore, treatment satisfaction is somehow controversially discussed in the field of healthcare research [49]. Another limitation is the cross-sectional design of this study. A longitudinal follow-up could not be conducted as the cohort size was too small and decreased too fast due to the sometimes rapidly progressive course of the disease. A longitudinal evaluation that additionally includes a closer consideration of caregivers’ needs can provide results that allow for more in-depth conclusions and should be performed in a future study. Another limitation was that the patients were at different stages of treatment at the timepoint of assessment and, unfortunately, analyzing subgroups was not possible due to the small cohort size. Furthermore, this study was conducted during a pandemic situation, and it is quite possible that the restrictions due to the pandemic influenced the assessed HR-QoL and treatment satisfaction.

Our study also had several advantages. This was the first cross-sectional study that investigated the correlations between several clinical aspects and HR-QoL/treatment satisfaction in a homogenous cohort of patients with malignant IDHwt gliomas who were treated along a predefined pathway within a high-volume dedicated academic brain tumor center. The field of caregiver satisfaction, which is sometimes underrepresented in research, was also considered.

## 5. Conclusions

In summary, we found in patients with IDHwt gliomas that a superior KPS was strongly associated with a higher level of functioning and a lower symptom burden. Although it was proven several times in other tumor entities that clinical factors such as sex, age, KPS and psychosocial distress have significant influences on the HR-QoL, there are only a few data available for patients with malignant IDHwt gliomas. Similarly, the literature shows that patients and caregivers act as a dyad for other tumor entities but there have been no studies that have investigated the HR-QoL and treatment satisfaction of caregivers of patients with malignant IDHwt gliomas. Therefore, this study was the first to address the caregivers and HR-QoL of patients with malignant IDHwt gliomas in more detail. In the future, an important extension of our study will be to verify our results in large multicentric patient cohorts and to identify clinical factors that significantly influence the HR-QoL and patient/caregiver treatment satisfaction of patients with malignant IDHwt gliomas to identify tools that support these patients and their caregivers at an early stage of the disease and throughout their entire disease course.

## Figures and Tables

**Table 1:** Demographic and clinical aspects of the investigated patient cohort (n = 61).

		Valid Number	Percent
Age groups at diagnosis	20.0–59.9	33	54.1%
	60.0–99.9	28	45.9%
Sex	Male	30	49.2%
	Female	31	50.8%
Tumor localization	Cerebrum	1	1.6%
	Frontal lobe	13	21.3%
	Temporal lobe	18	29.5%
	Parietal lobe	14	23.0%
	Occipital lobe	2	3.3%
	Brain, subareas overlapping	8	13.1%
	Brain, not specified	5	8.2%
Karnofsky Performance Scale at last QLQ-C30 assessment	100	1	1.6%
	90	22	36.1%
	80	20	32.8%
	70	12	19.7%
	60	3	4.9%
	50	3	4.9%
Epilepsy at first visit	No	28	45.9%
	Yes	33	54.1%
Range: diagnosis to last QLQ-C30 assessment	<18 months	33	54.1%
	≥18 months	28	45.9%
Psycho-oncological treatment	No	32	52.5%
	Yes	29	47.5%
Psychosocial distress at first visit *	No (<4)	37	60.7%
	Yes (≥4)	24	39.3%
	Total	61	100.0%

* Psychosocial distress at the first visit measured with the Hornheider Screening Instrument.

## Data Availability

The raw data supporting the conclusions of this article will be made available by the corresponding author on request.

## Supplementary Materials

The following supporting information can be downloaded at: https://www.mdpi.com/article/10.3390/curroncol31100459/s1, Figure S1: Consort flow of depicting the inclusion criteria (n = 61); Figure S2: Distribution of applied questionnaires during the study period (n = 61); Table S1: Comparison of mean scores of the functioning scales of the EORTC QLQ-C30 at last assessment of HR-QoL regarding the independent factors sex, age at diagnosis, Karnofsky Performance Scale and psychosocial distress (n = 61); Table S2: Comparison of mean scores of selected scales of the EORTC QLQ-BN20 at last assessment of HR-QoL regarding the independent factors sex, age at diagnosis, Karnofsky Performance Scale and psychosocial distress (n = 61); Table S3: Comparison of mean scores of the EORTC PATSAT-C33 scales for treatment satisfaction of patients at last assessment regarding the independent factors sex, age at diagnosis, Karnofsky Performance Scale and psychosocial distress (n = 61); Table S4: Comparison of mean scores of the EORTC PATSAT-C33 scales of treatment satisfaction of caregivers at last assessment regarding the independent factors sex, age at diagnosis, Karnofsky Performance Scale and psychosocial distress of the patients (n = 61).
