# Supplementary material for: Health-Related Quality of Life and Treatment Satisfaction of Patients with Malignant IDH Wild-Type Gliomas and Their Caregivers
Source: Curr Oncol. 2024 Oct 14;31(10):6155–70. doi: 10.3390/curroncol31100459 (PMC11506037; doi:10.3390/curroncol31100459)
Supplement: Supplementary file 1 [file curroncol-31-00459-s001.zip › curroncol-3227952-supplementary.pdf]

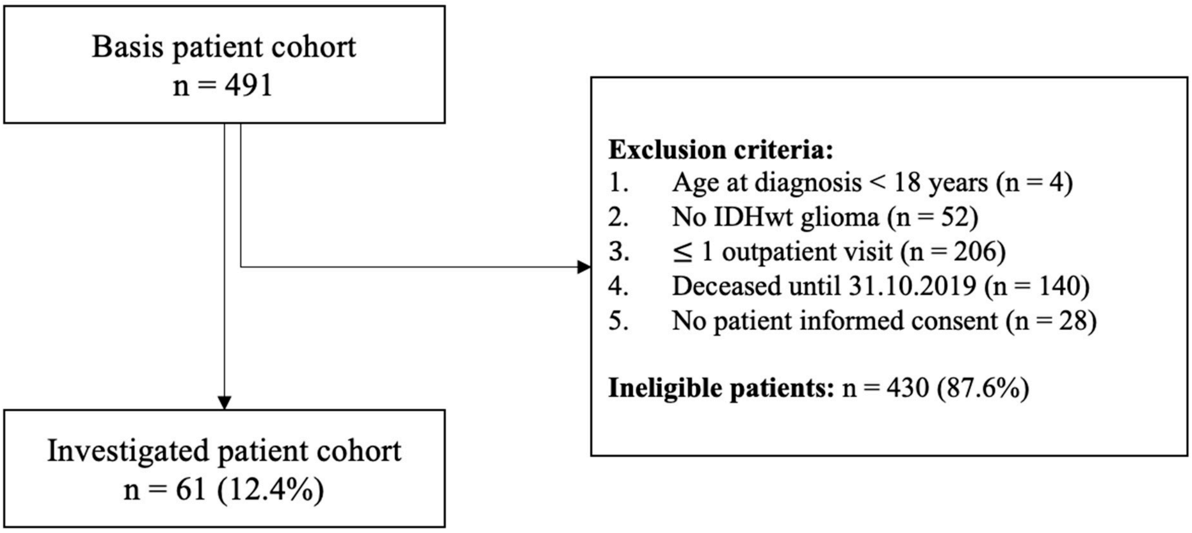

Figure S1: Consort flow of depicting the inclusion criteria (n = 61).

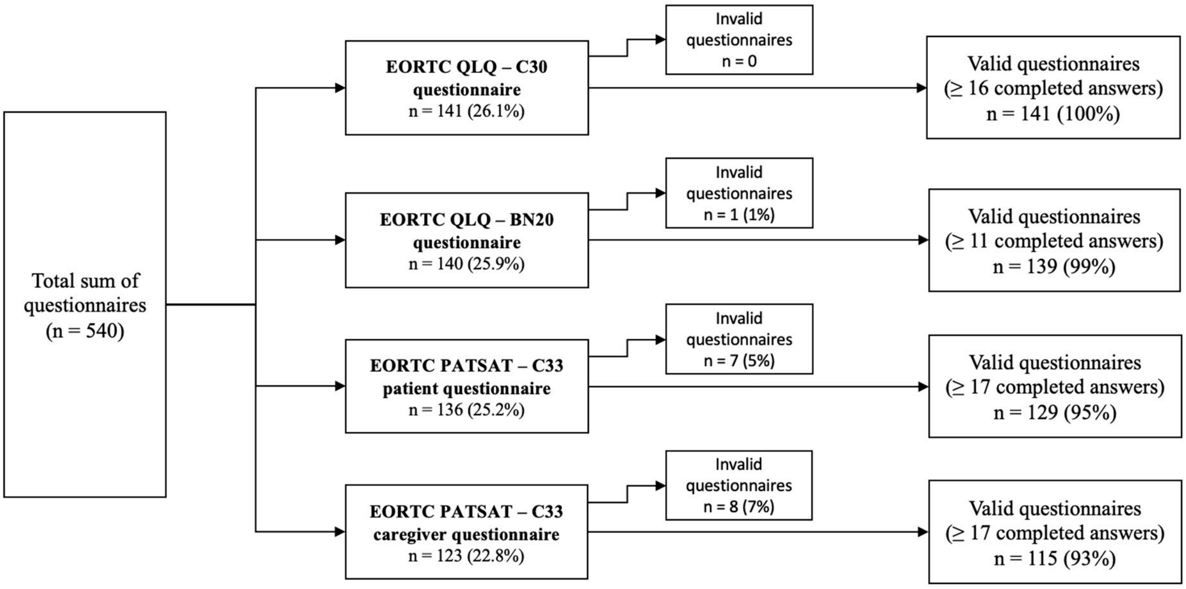

Figure S2: Distribution of applied questionnaires during the study period (n = 61).

Table S1: Comparison of mean scores of the functioning scales of the EORTC QLQ-C30 at last assessment of HR-QoL regarding the independent factors sex, age at diagnosis, Karnofsky Performance Scale and psychosocial distress (n = 61).

	Univariable linear regression analysis				Multiple linear regression analysis			
Functioning scale of EORTC QLQ-C30	*p*	t	B[#]	Adjusted R Square	*p*	t	B[#]	Adjusted R Square
**Sex: Female vs. male patients**								
Global health status	0.068	-1.858	-11.048	0.039	0.322	-0.998	-4.849	0.391
Physical functioning	0.003	-3.047	-24.222	0.123	**0.010**	**-2.680**	**-15.166**	**0.580**
Role functioning	0.030	-2.230	-18.978	0.062	0.131	-1.535	-8.892	0.594
Emotional functioning	0.695	0.394	2.706	-0.014	0.612	0.511	3.258	0.178
Cognitive functioning	0.170	-1.389	-10.556	0.015	0.499	-0.681	-4.324	0.350
Social functioning	0.079	-1.788	-15.753	0.035	0.494	-0.689	-5.559	0.292
**Age at diagnosis: patients ≥ 60 years old vs. < 60 years old**								
Global health status	0.327	-0.988	-6.016	<0.001	0.696	-0.393	-1.971	0.382
Physical functioning	0.579	0.558	4.792	0.012	0.900	0.126	0.910	0.580
Role functioning	0.310	-1.023	-9.019	0.001	0.837	-0.207	-1.221	0.591
Emotional functioning	0.203	-1.287	-8.754	0.011	0.361	-0.921	-5.894	0.189
Cognitive functioning	0.598	-0.530	-4.095	0.012	0.856	-0.183	-1.197	0.339
Social functioning	0.587	0.546	6.106	0.012	0.642	0.468	4.555	0.281
**Karnofsky Performance Scale[$]: increase per 10%**								
Global health status	<0.001	5.642	1.239	0.339	**<0.001**	**5.351**	**1.145**	**0.415**
Physical functioning	<0.001	8.438	2.151	0.543	**<0.001**	**8.074**	**1.942**	**0.616**
Role functioning	<0.001	7.281	2.085	0.464	**<0.001**	**7.632**	**1.912**	**0.618**
Emotional functioning	0.002	3.289	0.926	0.141	**0.004**	**3.002**	**0.868**	**0.153**
Cognitive functioning	<0.001	4.944	1.430	0.281	**<0.001**	**4.568**	**1.313**	**0.334**
Social functioning	<0.001	4.279	1.506	0.224	**<0.001**	**4.266**	**1.551**	**0.316**
**Psychosocial distress[*]: Patients with vs. without psychosocial distress**								
Global health status	0.014	-2.524	-15.015	0.082	0.144	-1.480	-7.490	0.391
Physical functioning	0.023	-2.335	-19.659	0.070	0.219	-1.242	-7.299	0.580
Role functioning	0.001	-3.348	-27.834	0.145	**0.013**	**-2.582**	**-15.475**	**0.594**
Emotional functioning	0.037	-2.137	-14.486	0.056	0.332	-0.978	-6.649	0.189
Cognitive functioning	0.033	-2.180	-16.573	0.059	0.355	-0.932	-6.167	0.350
Social functioning	0.036	-2.140	-19.088	0.056	0.598	-0.530	-4.905	0.292

Statistically significant results are marked as bold.

[#]B: Regression coefficient: positive coefficient represents a greater mean score of the scale. For the functioning scales, a greater mean score represents a better functioning status.

[*]Psychosocial distress, measured with the Hornheider Screening Instrument, at first outpatient visit.

[$]Karnofsky Performance Scale at the time of last assessment of health-related quality of life.

Table S2: Comparison of mean scores of selected scales of the EORTC QLQ-BN20 at last assessment of HR-QoL regarding the independent factors sex, age at diagnosis, Karnofsky Performance Scale and psychosocial distress (n = 61).

Scale of EORTC QLQ-BN20	Univariable linear regression analysis				Multiple linear regression analysis			
	p	t	B#	Adjusted R Square	*p*	t	B#	Adjusted R Square
**Sex: Female vs. male patients**								
Future uncertainty	0.056	-1.949	-14.731	0.045	0.230	-4.784	-1.399	0.333
Visual disorder	0.653	-0.452	-2.874	0.014	0.207	-1.276	-7.831	0.174
Motor dysfunction	0.023	2.341	19.651	0.071	0.171	1.387	8.334	0.552
Communication deficit	0.310	-1.024	-9.642	0.001	0.773	-0.290	-2.118	0.443
**Age at diagnosis: patients ≥ 60 years old vs. < 60 years old**								
Future uncertainty	0.930	-0.088	-0.685	0.017	0.656	-0.448	-2.993	0.323
Visual disorder	0.077	1.801	11.228	0.037	0.351	0.941	5.790	0.164
Motor dysfunction	0.899	-0.127	-1.122	0.017	0.440	-0.779	-4.855	0.549
Communication deficit	0.002	3.248	28.499	0.137	**<0.001**	**3.542**	**25.774**	**0.452**
**Karnofsky Performance Scale$: increase per 10%**								
Future uncertainty	<0.001	-5.353	-1.534	0.315	**<0.001**	**-4.784**	**-1.399**	**0.333**
Visual disorder	0.088	-1.737	-0.491	0.034	0.187	-1.335	-0.369	0.164
Motor dysfunction	<0.001	-8.094	-2.222	0.522	**<0.001**	**-7.279**	**-2.029**	**0.552**
Communication deficit	<0.001	-3.890	-1.477	0.191	**<0.001**	**-4.034**	**-1.330**	**0.407**
**Psychosocial distress*: Patients with vs. without psychosocial distress**								
Future uncertainty	0.063	1.895	14.677	0.041	0.164	1.411	9.236	0.333
Visual disorder	<0.001	3.597	21.032	0.171	**0.019**	**2.426**	**17.117**	**0.164**
Motor dysfunction	0.018	2.442	20.833	0.078	0.052	1.988	12.030	0.552
Communication deficit	0.005	2.945	26.727	0.113	0.207	1.276	9.816	0.452

Statistically significant results are marked as bold.

#B: Regression coefficient: positive coefficient represents a greater mean score of the scale. A greater mean score represents a higher symptom burden.

*Psychosocial distress, measured with the Hornheider Screening Instrument, at first outpatient visit.

$Karnofsky Performance Scale at the time of last assessment of health-related quality of life.

Table S3: Comparison of mean scores of the EORTC PATSAT-C33 scales for treatment satisfaction of patients at last assessment regarding the independent factors sex, age at diagnosis, Karnofsky Performance Scale and psychosocial distress (n = 61).

	Univariable linear regression analysis				Multiple linear regression analysis			
Scale of EORTC PATSAT-C33	*p*	t	B[#]	Adjusted R Square	*p*	t	B[#]	Adjusted R Square
**Sex: Female vs. male patients**								
Doctor/ technical skills	0.241	-1.186	-7.314	0.007	0.463	-0.739	-4.491	0.072
Doctor/ information exchange	0.192	-1.320	-8.231	0.012	0.424	-0.805	-4.928	0.090
Doctor/ affective behavior	0.107	-1.638	-0.210	0.028	0.237	-1.196	-6.521	0.098
Family involvement	0.282	-1.086	-6.810	0.003	0.423	-0.807	-4.832	0.104
Overall care	0.249	-1.165	-6.618	0.006	0.458	-0.747	-4.178	0.072
**Age at diagnosis: patients ≥ 60 years old vs. < 60 years old**								
Doctor/ technical skills	0.032	-2.193	-13.215	0.061	0.081	-1.774	-10.926	0.080
Doctor/ information exchange	0.397	-0.852	-5.387	0.005	0.850	-0.189	-1.202	0.061
Doctor/ affective behavior	0.093	-1.709	-9.512	0.032	0.387	-0.873	-4.927	0.098
Family involvement	0.006	-2.847	-16.987	0.109	**0.010**	**-2.659**	**-16.070**	**0.102**
Overall care	0.135	-1.516	-8.586	0.022	0.424	-0.805	-4.620	0.079
**Karnofsky Performance Scale[$]: increase per 10%**								
Doctor/ technical skills	0.978	0.028	0.008	0.017	0.804	0.249	0.068	0.064
Doctor/ information exchange	0.920	0.101	0.029	0.017	0.792	0.264	0.074	0.074
Doctor/ affective behavior	0.612	0.510	0.128	0.013	0.648	0.459	0.121	0.085
Family involvement	0.485	-0.703	-0.195	0.009	0.453	-0.755	-0.198	0.102
Overall care	0.804	0.249	0.064	0.016	0.718	0.363	0.094	0.065
**Psychosocial distress[*]: Patients with vs. without psychosocial distress**								
Doctor/ technical skills	0.363	0.918	5.846	0.003	0.782	0.278	1.975	0.065
Doctor/ information exchange	0.225	-1.225	-7.873	0.008	0.899	-0.128	-0.905	0.074
Doctor/ affective behavior	0.036	-2.149	-12.074	0.058	0.359	-0.925	-5.877	0.098
Family involvement	0.739	0.335	2.174	0.016	0.245	1.176	7.392	0.115
Overall care	0.028	-2.250	-12.750	0.064	0.268	-1.118	-7.260	0.079

Statistically significant results are marked as bold.

[#]B: Regression coefficient: positive coefficient represents a greater mean score of the scale. A greater score means a higher level of treatment satisfaction with perceived care quality.

[*]Psychosocial distress, measured with the Hornheider Screening Instrument, at first outpatient visit.

[$]Karnofsky Performance Scale at the time of last assessment of treatment satisfaction.

Table S4: Comparison of mean scores of the EORTC PATSAT-C33 scales of treatment satisfaction of caregivers at last assessment regarding the independent factors sex, age at diagnosis, Karnofsky Performance Scale and psychosocial distress of the patients (n = 61).

	Univariable linear regression analysis				Multiple linear regression analysis			
Scale of EORTC PATSAT-C33	*p*	t	B#	Adjusted R Square	*p*	t	B#	Adjusted R Square
**Sex: Female vs. male patients**								
Doctor/ technical skills	0.269	-1.119	-6.643	0.005	0.392	-0.865	-5.088	0.062
Doctor/ information exchange	0.191	-1.326	-8.213	0.015	0.247	-1.174	-7.055	0.110
Doctor/ affective behavior	0.907	0.117	0.724	0.019	0.991	0.012	0.072	0.016
Family involvement	0.622	-0.496	-3.148	0.015	0.624	-0.493	-3.200	0.036
Overall care	0.696	0.394	2.000	0.017	0.690	0.401	2.077	0.037
**Age at diagnosis: patients ≥ 60 years old vs. < 60 years old**								
Doctor/ technical skills	0.200	-1.299	-7.711	0.014	0.647	-0.460	-2.887	0.067
Doctor/ information exchange	0.356	-0.931	-5.844	0.003	0.898	-0.129	-0.836	0.090
Doctor/ affective behavior	0.410	-0.831	-5.154	0.006	0.470	-0.728	-4.453	0.026
Family involvement	0.977	0.029	0.187	0.020	0.960	0.050	0.326	0.036
Overall care	0.646	-0.462	-2.709	0.015	0.917	-0.105	-0.549	0.037
**Karnofsky Performance Scale of the patients$: increase per 10%**								
Doctor/ technical skills	0.727	0.351	0.092	0.018	0.954	0.059	0.016	0.046
Doctor/ information exchange	0.423	-0.808	-0.221	0.007	0.199	-1.304	-0.361	0.123
Doctor/ affective behavior	0.775	-0.288	-0.079	0.018	0.919	-0.102	-0.027	0.017
Family involvement	0.835	-0.210	-0.058	0.019	0.733	-0.344	-0.100	0.055
Overall care	0.985	0.018	0.004	0.020	0.930	0.088	0.021	0.058
**Psychosocial distress*: Patients with vs. without psychosocial distress**								
Doctor/ technical skills	0.014	-2.538	-14.583	0.100	0.069	-1.859	-12.931	0.067
Doctor/ information exchange	0.056	-1.957	-12.083	0.055	0.094	-1.710	-12.160	0.110
Doctor/ affective behavior	0.248	-1.168	-7.244	0.007	0.210	-1.270	-7.728	0.046
Family involvement	0.237	-1.196	-7.642	0.008	0.236	-1.201	-8.173	0.026
Overall care	0.661	-0.442	-2.273	0.016	0.643	-0.466	-2.541	0.053

Statistically significant results are marked as bold.

#B: Regression coefficient: positive coefficient represents a greater mean score of the scale. A greater score means a higher level of treatment satisfaction with perceived care quality.

*Psychosocial distress, measured with the Hornheider Screening Instrument, at first outpatient visit of the patients.

$Karnofsky Performance Scale of the patients at the time of last assessment of treatment satisfaction.
